# Benefits of a Pre-Treatment Comprehensive Geriatric Assessment in a Rare Case of *Gemella Haemolysans* Endocarditis in an 86-Year-Old Patient and a Review of the Literature

**DOI:** 10.3390/medicina55060292

**Published:** 2019-06-20

**Authors:** Lucie Sadaune, Frédéric Roca, Mathilde Bordage, Vincent Le Guillou, Anais Lesourd, Anne Michel

**Affiliations:** 1 Department of Geriatric Medicine, Rouen University Hospital, 1 rue de Germont, 76000 Rouen, France; lucie.sadaune@chu-rouen.fr (L.S.); frederic.roca@chu-rouen.fr (F.R.); mathilde.bordage@chu-rouen.fr (M.B.); 2 Department of Thoracic and Cardiac Surgery, Rouen University Hospital, 1 Rue de Germont, 76000 Rouen, France; vincent.le-guillou@chu-rouen.fr; 3 Department of Infectious Disease, Rouen University Hospital, 1 Rue de Germont, 76000 Rouen, France; anais.lesourd@chu-rouen.fr; 4 Department of Geriatric Medicine, Evangelisches Krankenhaus Kalk, Buchforststraße 2, 51103 Cologne, Germany

**Keywords:** infective endocarditis, elderly, *Gemella haemolysans*, comprehensive geriatric assessment

## Abstract

Infective endocarditis is a serious condition, which is associated with high mortality in elderly patients. *Gemella haemolysans* (*GH*) is a microorganism from the *Streptococcus* family, rarely involved in infective endocarditis. Here, we present a case of *Gemella haemolysans* endocarditis in an 86-year-old patient, successfully treated by antibiotics and surgery following a pre-treatment comprehensive geriatric assessment (CGA). This case is discussed in the context of a review of all published cases of *Gemella haemolysans* endocarditis. We illustrate the benefit of a systematic pre-treatment comprehensive geriatric assessment in elderly patients with infective endocarditis.

## 1. Introduction

*Gemella haemolysans* (*GH*) is a microorganism from the *Streptococcus* family, usually sensible to penicillin and cephalosporin, rarely involved in infective endocarditis. The endocarditis in-hospital mortality rate ranges from 15 to 30% [1] and old age is a well-known predictor of a poor prognostic. A comprehensive geriatric assessment (CGA) is a multidimensional interdisciplinary diagnostic process aimed at evaluating and optimizing physical, psychological, functional, and social issues in the older population. It is implemented to develop a coordinated and integrated treatment strategy seeking to improve long-term outcomes. In acute geriatric departments, a CGA is routinely performed for every patient at the time of admission. A multidisciplinary team including medical, nursing, and therapy staff as well as social workers is necessary. Different scores to assess social network, comorbidities, cognition, mobility, nutrition, and frailty are used. A CGA has already proven to be beneficial in various diseases such as cancer [2] and hip fracture [3].

Here, we present a case of *GH* endocarditis in an 86-year-old patient successfully treated by antibiotics and surgery. We also review all cases of *GH* endocarditis previously published in the literature. Moreover, we illustrate the benefits of a systematic pre-treatment CGA and of multidisciplinary management involving the infectious disease, surgery, and geriatric departments in a very old patient with infective endocarditis.

## 2. Case Report

An 86-year-old woman was admitted to the University Hospital of Rouen with suspected pneumonia and persistent fever showing no improvement after four days of treatment with amoxicillin/clavulanic acid. She had a history of hypertension, hypercholesterolemia, moderated aortic stenosis (mean gradient of 25 mmHg and aortic valve area 1 cm^2^) and stable ischemic heart disease (stent in the circonflex artery 2015). On admission, she was afebrile and without other symptoms. A physical examination found no sign of endocarditis (no systolic murmur was heard). White blood cell count was 15.5 giga/L with 81.7% of neutrophils and C-reactive protein 141 mg/L. Two sets of blood culture were positive for *GH* (Penicillin minimum inhibitory concentration was 0.125 mg/L). An intravenous treatment with amoxicillin (12 g/day) and gentamicin (200 mg/day) was started under the hypothesis of infective endocarditis. After five days, the patient’s condition severely deteriorated due to acute pulmonary edema. The transthoracic echocardiography found a severe aortic regurgitation without vegetation (vena contracta measured 6 mm and the ratio jet width on left ventricular outflow tract (LVOT) was 100%), the left ventricle was not dilated and non-hypertrophic and the ejection fraction was 60%, which was normal. A comprehensive geriatric assessment (CGA) had been performed on admission to screen for geriatric conditions that might influence the prognosis and thus therapeutic strategies. The Charlson Comorbidity Index was 5 indicating a low degree of comorbidities, the functional abilities evaluated by the Instrumental Activities of Daily Living (IADL) and the Activities of Daily Living (ADL) were normal. There was no sign or history of cognitive disorders and no sign of delirium confirmed by a negative Confusion Assessment Method (CAM). Concerning the nutritional parameters, the patient was overweight (BMI = 29.4 kg/m^2^) with a hypoalbuminaemia (21 g/L). The combination of absence of major geriatric syndromes and the severity of the valvular disease supported a potential benefit of a surgical approach. The patient was transferred to the department of cardiology on the day of the transthoracic echocardiography and underwent aortic valvular replacement surgery by a bioprothesis two days later. This resulted in an improvement in both clinical and blood values following surgery. The anatomo-pathological examination of the native valve found a Gram-positive coccus. The polymerase chain reaction (PCR) 16S rRNA gene amplification sequencing of the native valve confirmed the existence of bacterial material. The study of the amplified sequence confirmed the presence of *GH*. Abdominal and pelvic CT and a dental exam did not show any anomalies. The antibiotic therapy was continued for six weeks. The patient went to a rehabilitation ward to undergo physiotherapy and an oral nutrition program. She went back home in the same conditions as before the hospitalization. Albumin four months after discharge from hospital was 35 g/L.

The patient on whom we wrote the case report signed an informed consent form to approve the use of her data. An ethical approval from the ethical committee of our hospital was not necessary according to the GCP guidelines of our university.

## 3. Discussion

*GH* was described for the first time in 1938 as *Neisseria haemolysans* [4]. It was reclassified in 1960 into a new genus, *Gemella*, after Reyn et al. demonstrated biochemical differences (i.e., catalase and oxidase negative reactions) compared to *Neisseria* [5]. *GH* is easily decolorized during Gram staining which can lead to a Gram-variable or even a Gram-negative coloration. For this reason, it can be misidentified or stay unidentified. The bacterium is commonly found in the oropharynx, the upper airways, and the digestive tract. Thus, dental infection and neoplastic lesion from the digestive tract can be responsible for *GH* bacteremia [6].

According to our knowledge, only 24 documented cases of *GH* endocarditis have been reported until now including our case [6,7,8,9,10,11,12,13,14,15,16,17,18,19,20,21,22,23,24,25]. They have been summarized in Table 1. The mean age of these cases was 56 years (ranged from 20 to 87 years) and 60.8% of patients had an aortic valve localization. In this case, we report the oldest patient with *GH* endocarditis that survived after undergoing an early surgical treatment. Only 11 of the 23 patients had a surgical valve replacement, whereas its benefit has been proved in a randomized trial compared to conventional treatment with significant reduction of embolic events and hospital death [26]. The presence of heart failure caused by severe aortic insufficiency is one of the main indications for urgent surgery [1]. This was also the case with our patient. While planned cardiac surgery in the population of patients aged 80 years or older leads to a higher mortality rate and increased risk of dependency [27], our pre-operative CGA did not identify any major geriatric contraindications to surgery. Apart from hypoalbuminemia, which can be associated with undernourishment and inflammatory syndrome, the CGA revealed that our patient was a robust elderly person despite her advanced age.

The CGA is a multidimensional interdisciplinary diagnostic process for evaluating and optimizing physical, psychological, functional, and social issues of the older population. It is implemented to develop a coordinated and integrated plan for treatment and to improve long-term outcomes. The method has proven to be beneficial in terms of mortality, institutionalization [28], postoperative complications, and length of stay compared to usual care in scheduled cardiac and non-cardiac surgery [29]. Additionally, a CGA evaluates conditions such as frailty, which are not usually screened by cardiac surgery scores [30] and can affect the prognosis. Until now, the positive effect of a CGA before cardiac surgery as treatment of infective endocarditis has not been investigated. The main reason is probably the emergency nature of such a situation. However, a CGA has already demonstrated a positive effect on outcomes in urgent surgery such as hip fracture [3]. Thus, we hypothesize that a pre-treatment CGA has the potential to improve outcomes of infective endocarditis treatment in the elderly population.

## 4. Conclusions

Our case illustrates the major role of geriatricians and a CGA in decisions concerning invasive treatment for elderly patients in emergency situations such as infective endocarditis. Thus, geriatricians should systematically be involved in the decisions of the endocarditis team when elderly patients are concerned. Further studies are required to evaluate the positive impact of a pre-treatment CGA on outcomes of infective endocarditis in the elderly.

## Figures and Tables

**Table 1:** Cases of *Gemella haemolysans* endocarditis reported in the literature.

Author	Year	Sex/Age	Valve	Antibiotherapy	Surgery	Evolution	Source of Infection
Chatelain R. et al. [7]	1982	M/62	Mitral	Cefamandole and gentamicin, change to penicillin, change to oral amoxicillin for 2 months	No	Favorable	Dental
Chatelain R. et al. [7]	1982	M/48	Aortic	Penicillin and streptomycin during 40 days	Yes	Favorable	Dental
Chatelain R. et al. [7]	1982	M/56	Mitral	Penicillin and gentamicin for 6 weeks	No	Favorable	Dental
Laudat P. et al. [8]	1984	M/68	Aortic	Penicillin and streptomycin for 40 days	Yes	Favorable	Dental
Kaufhold A. et al. [9]	1989	F/62	Mitral	Penicillin during 37 days and tobramycin during 19 days	No	Favorable	No
Morea P. et al. [10]	1991	M/52	Aortic valvular prosthesis	Erythromycin and rifampicin	Yes	Favorable	No
Brack M.J. et al. [11]	1991	M/74	Mitral	Penicillin, gentamicin during 2 weeks, change to oral amoxicillin for 4 weeks and intramuscular gentamicin for 2 weeks	No	Favorable	No
Frésard A. et al. [12]	1993	M/42	Aortic	Vancomycin and fusidic acid for 10 days, change to amoxicillin and gentamicin for 2 weeks	No	Favorable	Dental
Helft G. et al. [6]	1993	M/71	Mitral	Amoxicillin-clavulanic acid and gentamicin	No	Favorable	Colorectal cancer
Devuyst O. et al. [13]	1993	M/53	Mitral	Penicillin and gentamicin	No	Favorable	No
Matsis P. et al. [14]	1994	M/20	Aortic	Penicillin and gentamicin for 4 weeks	Yes	Favorable	No
Samuel L. et al. [15]	1995	M/34	Aortic valvular prosthesis	Cefuroxime and tobramycin during 14 days, change to ciprofloxacin and erythromycin	No	Favorable	Dental
La Scola B. et al. [16]	1998	M/63	Mitral	Amoxicillin, amikacin during 3 weeks	Yes	Favorable	No
La Scola B. et al. [16]	1998	M/74	Aortic	Amoxicillin during 3 weeks and gentamicin during 1 week	Yes	Favorable	No
Zingaro L. et al. [17]	1999	M/49	Aortic	Piperacillin and ciprofloxacin	Yes	Favorable	No
Mosquera J.D. et al. [18]	2000	M/77	Aortic	Penicillin and tobramycin during 2 weeks	No	Favorable	No
Kim Y.C. et al. [19]	2000	M/37	Mitral	Vancomycin, gentamicin, ceftriaxone	Yes	Favorable	No
Khan R. et al. [20]	2004	M/80	Mitral	Levofloxacin, change to vancomycin and rifampicin, change to ampicillin and gentamicin	No	Favorable	Dental
Ramchandani M.S. et al. [21]	2014	F/40	Aortic valvular prosthesis	Vancomycin, ceftriaxone, gentamicin, and rifampicin, change to ampicillin and gentamicin for 4 weeks	Yes	Favorable	No
Liu D. et al. [22]	2016	F/87	Aortic	Ampicillin and gentamicin during 4 weeks	No	Death	No
Quaesaet L. et al. [23]	2016	M/39	Aortic valvular prosthesis	Amoxicillin and rifampicin during 45 days	No	Favorable	Dental
Winkler J. et al. [24]	2016	M/67	Mitral	Unknown antibiotherapy during 6 weeks	No	Death	No
Ando A. et al. [25]	2016	M/24	Aortic/mitral	Penicillin and vancomycin during 7 weeks	Yes	Favorable	Dental
Our case	2019	F/86	Aortic	Amoxicillin and clavulanic acid, change to amoxicillin and gentamicin during 6 weeks	Yes	Favorable	No
